# Diagnosing sepsis is subjective and highly variable: a survey of intensivists using case vignettes

**DOI:** 10.1186/s13054-016-1266-9

**Published:** 2016-04-06

**Authors:** Chanu Rhee, Sameer S. Kadri, Robert L. Danner, Anthony F. Suffredini, Anthony F. Massaro, Barrett T. Kitch, Grace Lee, Michael Klompas

**Affiliations:** Department of Population Medicine, Harvard Medical School and Harvard Pilgrim Health Care Institute, 401 Park Drive, Suite 401, Boston, MA USA; Department of Medicine, Brigham and Women’s Hospital, Boston, MA USA; Critical Care Medicine Department, Clinical Center, National Institutes of Health, Bethesda, MD USA; Department of Medicine, North Shore Medical Center, Salem, MA USA

## Abstract

**Background:**

Sepsis is the focus of national quality improvement programs and a recent public reporting measure from the Centers for Medicare and Medicaid Services. However, diagnosing sepsis requires interpreting nonspecific signs and can therefore be subjective. We sought to quantify interobserver variability in diagnosing sepsis.

**Methods:**

We distributed five case vignettes of patients with suspected or confirmed infection and organ dysfunction to a sample of practicing intensivists. Respondents classified cases as systemic inflammatory response syndrome, sepsis, severe sepsis, septic shock, or none of the above. Interobserver variability was calculated using Fleiss’ κ for the five-level classification, and for answers dichotomized as severe sepsis/septic shock versus not-severe sepsis/septic shock and any sepsis category (sepsis, severe sepsis, or septic shock) versus not-sepsis.

**Results:**

Ninety-four physicians completed the survey. Most respondents (88 %) identified as critical care specialists; other specialties included pulmonology (39 %), anesthesia (19 %), surgery (9 %), and emergency medicine (9 %). Respondents had been in practice for a median of 8 years, and 90 % practiced at academic hospitals. Almost all respondents (83 %) felt strongly or somewhat confident in their ability to apply the traditional consensus sepsis definitions. However, overall interrater agreement in sepsis diagnoses was poor (Fleiss’ κ 0.29). When responses were dichotomized into severe sepsis/septic shock versus not-severe sepsis/septic shock or any sepsis category versus not-sepsis, agreement was still poor (Fleiss’ κ 0.23 and 0.18, respectively). Seventeen percent of respondents classified one of the five cases as severe sepsis/septic shock, 27.7 % rated two cases, 33.0 % respondents rated three cases, 19.2 % rated four cases, and 3.2 % rated all five cases as severe sepsis/septic shock. Among respondents who felt strongly confident in their ability to use sepsis definitions (*n* = 45), agreement was no better (Fleiss’ κ 0.28 for the five-category classification, and Fleiss’ κ 0.21 for the dichotomized severe sepsis/septic shock classification). Cases were felt to be extremely or very realistic in 74 % of responses; only 3 % were deemed unrealistic.

**Conclusions:**

Diagnosing sepsis is extremely subjective and variable. Objective criteria and standardized methodology are needed to enhance consistency and comparability in sepsis research, surveillance, benchmarking, and reporting.

**Electronic supplementary material:**

The online version of this article (doi:10.1186/s13054-016-1266-9) contains supplementary material, which is available to authorized users.

## Background

Sepsis, the syndrome of dysregulated inflammation that occurs with severe infection, is associated with high morbidity, mortality, and cost [1, 2]. The devastating toll of sepsis on society has prompted national performance improvement initiatives and governmental mandates for sepsis care and reporting, including a recent quality measure issued by the Centers for Medicare and Medicaid Services (CMS) [3–5]. However, reliably identifying cases of sepsis and septic shock is complicated because there is no gold standard diagnostic test [6]. The diagnosis therefore requires clinicians to interpret a constellation of nonspecific physiological and laboratory abnormalities among patients with suspected or definite infection [7, 8]. To make the diagnosis of severe sepsis, clinicians must decide whether a patient has an infection, whether acute organ dysfunction is present, and whether acute organ dysfunction (when present) is attributable to infection. These determinations can be subjective and it is thus highly conceivable that thoughtful clinicians might differ substantially in their judgments.

Variability in how clinicians diagnose sepsis has important implications for clinical care, epidemiologic and clinical studies, public health surveillance, pay-for-performance initiatives, and quality improvement programs. Our aim in the present study was to evaluate whether and to what degree intensivists agree in how they diagnose sepsis. To do so, we distributed case vignettes of common scenarios of patients with suspected infection and organ dysfunction to a sample of intensivists. We hypothesized that there would be significant variability in sepsis diagnoses, and that this variability would exist independent of physicians’ degree of confidence in their ability to apply the traditional consensus definitions of sepsis.

## Methods

### Survey and case vignette description

We designed a survey that began with several background questions aimed at gaining an understanding of the characteristics of the responding clinician, including years of clinical experience, specialty, volume of intensive care unit (ICU) patients seen on a regular basis, type of hospital practice, and baseline level of confidence in the clinician’s knowledge and ability to apply the international consensus clinical definitions of sepsis. Five case vignettes were then shown that described patients with suspected or documented infection and signs suggestive of organ dysfunction (Additional file 1: Appendix 1). The vignettes were designed with input from infectious disease and critical care specialists to replicate scenarios commonly seen in routine clinical practice. Four of the five cases had negative blood cultures, approximating the frequency of documented bacteremia among patients with severe sepsis [9]. Case A described a patient with suspected pneumonia and a prior history of congestive heart failure who developed shock and respiratory failure. Case B described a patient who presented with pyelonephritis and acute kidney injury. Case C described a patient with diarrhea caused by colitis who presented with hypotension. Case D described a patient with a severe chronic obstructive pulmonary disease exacerbation requiring intubation. Case E was designed to be an unequivocal case of septic shock with gram-negative rod bacteremia leading to shock, multiorgan failure, and death, so as to serve as a “control” case to ensure respondents were attentive to the cases and reasonably knowledgeable about sepsis definitions. We chose to describe both the initial presentation and the subsequent hospital course in all the cases because our primary interest was in whether variability in diagnosing sepsis would exist even after patients’ clinical courses were clear, rather than focusing solely on the initial undifferentiated phase of illness at presentation. Furthermore, this approach approximates the process used for sepsis coding and quality reporting.

Responders were then asked to classify whether each patient had systemic inflammatory response syndrome (SIRS), sepsis, severe sepsis, septic shock, or none of the above, accompanied by a free text space to explain their choice. Respondents were also asked about their level of confidence in their diagnosis (ordinal scale 1–5) and how realistic and representative the cases were of actual patients they had seen (ordinal scale 1–5). The survey was piloted and refined among a small group of physicians before dissemination.

### Survey distribution method and target groups

After obtaining approval from the Harvard Pilgrim Health Care Institute Institutional Review Board (protocol 657743-4), we electronically distributed the survey using SurveyMonkey (SurveyMonkey.com, LLC, Palo Alto, CA, USA) to two groups: (1) physicians in the U.S. Critical Illness and Injury Trials Group (USCIITG) and (2) all attending intensivists in the medical ICU or surgical ICU at four hospitals in the Boston area (Massachusetts General Hospital, Brigham and Women’s Hospital, Brigham and Women’s Faulkner Hospital, and North Shore Hospital). The USCIITG is a national group of clinical researchers—primarily intensivists—who collaborate on critical care research projects. We chose to focus on intensivists because severe sepsis is most commonly treated in ICUs, and we focused on attending physicians (rather than trainees) to minimize any potential confounding due to lack of experience. Emails were sent to members of the USCIITG listserv (*n* = 477) and to each intensivist affiliated with the four Boston hospitals (*n* = 98). The online survey period took place from September through November 2015. In addition, intensivists who attended the annual USCIITG conference in Bethesda, MD, USA (16–17 November 2015), and who had not already completed the online survey were handed paper surveys. Respondents were told the purpose of the study was to examine how physicians apply sepsis diagnoses to patients and were offered a $15 gift card as a token of appreciation for completing the survey.

### Statistical analyses

We compared interobserver variation in sepsis diagnoses among participants using Fleiss’ κ statistic, a common metric for quantifying agreement among multiple raters for categorical ratings [10]. We analyzed interobserver agreement among the five-level classification (SIRS, sepsis, severe sepsis, septic shock, or none of the above); however, we also dichotomized answers into severe sepsis/septic shock or not, as this distinction is more relevant for purposes of quality reporting, clinical trials, and epidemiologic studies. We further dichotomized answers into any sepsis category (sepsis, severe sepsis, or septic shock) or not (SIRS or none of the above) to account for the possibility that some clinicians may not necessarily differentiate sepsis from severe sepsis. As suggested by Fleiss et al and prior authors, we considered κ values greater than 0.75 to represent strong agreement, values between 0.40 and 0.75 to be fair to good agreement, and values less than 0.40 to be poor agreement [11, 12]. We also performed a subgroup analysis limited to respondents who felt strongly confident (5 on a scale of 5) in their ability to describe and use the consensus sepsis definitions. When available, we examined the free text explanations given for the choice of sepsis diagnoses for each case and summarized them into several categories defined a priori. Because each case described possible infection and organ dysfunction, we were interested mainly in understanding why respondents labeled cases as anything other than severe sepsis/septic shock. Fleiss’ κ analysis was performed using an online software package [13]. All other analyses were conducted using SAS version 9.4 software (SAS Institute, Cary, NC, USA).

## Results

### Survey respondent characteristics

Of approximately 575 physicians contacted, 94 (16.8 %) completed the entire survey. Most respondents completed the survey electronically via SurveyMonkey (*n* = 78) rather than on paper (*n* = 16). The characteristics of the survey respondents are summarized in Table 1. The majority (88.3 %) of respondents identified one of their subspecialties as critical care; other common subspecialties were pulmonology, anesthesia, surgery, and emergency medicine. Most respondents (66.0 %) were from the northeastern United States and practiced in academic hospitals (90.4 %). Most were strongly confident (47.9 %) or somewhat confident (35.1 %) in their ability to describe and use the international consensus clinical definitions of sepsis; only 7.4 % reported not feeling confident.

**Table 1:** Survey respondent characteristics (*N* = 94 respondents)

Respondent profile	Data
Subspecialties, *n* (%)	
Critical care	83 (88.3 %)
Pulmonary	37 (39.4 %)
Anesthesia	18 (19.1 %)
Surgery	8 (8.5 %)
Emergency medicine	8 (8.5 %)
Infectious diseases	4 (4.3 %)
Academic hospital (versus community hospital), *n* (%)	85 (90.4 %)
Region, *n* (%)	
Northeastern United States	62 (66.0 %)
Midwestern United States	13 (13.8 %)
Southern United States	8 (8.5 %)
Western United States	10 (10.6 %)
Non-U.S. country	1 (1.1 %)
Median years in practice (IQR)	8 (3–15)
Median percentage of time doing clinical work (IQR)	50 (25–80)
Median percentage of clinical time spent in ICU (IQR)	50 (30–90)
Median number of ICU patients cared for each month (IQR)	40 (20–55)

*ICU* intensive care unit, *IQR* interquartile range

### Agreement in diagnosing sepsis

**Fig. 1:**
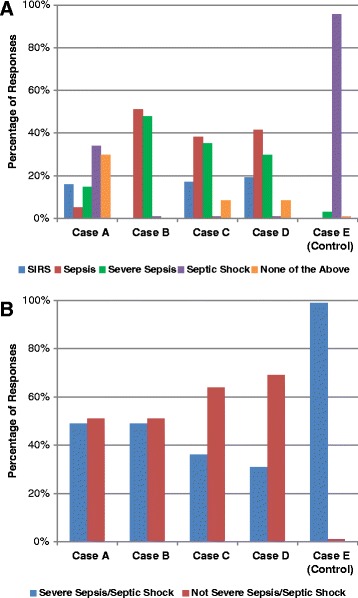
Distribution of responses for each case for (**a**) five-level classifications (systemic inflammatory response syndrome, sepsis, severe sepsis, septic shock, or none) and (**b**) dichotomized classification (severe sepsis/septic shock or not). *SIRS* systemic inflammatory response syndrome

Overall interrater agreement with respect to the five-level classification (SIRS, sepsis, severe sepsis, septic shock, or none of the above) in the five cases was poor, with a Fleiss’ κ of 0.29. Aside from the “control” case (case E), there was a wide range in the diagnoses assigned to the cases (Fig. 1a). This was particularly noticeable for case A (suspected pneumonia, heart failure, respiratory failure, and shock), as 34.0 % of respondents diagnosed septic shock and 14.9 % diagnosed severe sepsis, yet 29.8 % diagnosed “none of the above,” 16.0 % diagnosed SIRS, and 5.3 % diagnosed sepsis alone. Agreement in the control case E (bacteremic septic shock leading to multiorgan failure and death), however, was excellent, as 90 (95.7 %) of 94 respondents labeled the case as septic shock and 3 respondents (3.2 %) labeled the case as “severe sepsis”; 1 respondent (1.1 %) labeled the case as “none of the above.” When analyzing only cases A–D, agreement among respondents was nearly random (κ 0.11).

When sepsis classifications were dichotomized into severe sepsis (including septic shock) or not, agreement was no better (κ 0.23) than with the five-level classification. For cases A and B, there was a nearly even split on whether respondents believed the patients had severe sepsis/septic shock. Specifically, severe sepsis/septic shock was diagnosed by 48.9 % of respondents for case A, 48.9 % for case B, 36.2 % for case C, 30.9 % for case D, and 98.9 % for case E (Fig. 1b). Overall, 17.1 % of respondents rated only one of the cases as severe sepsis/septic shock, 27.7 % rated two cases, 33.0 % respondents rated three cases, 19.2 % rated four cases, and 3.2 % rated all five cases as severe sepsis/septic shock (median 3 cases, interquartile range 2–3). When we dichotomized responses into any sepsis category (sepsis, severe sepsis, septic shock) versus not-sepsis, agreement was still poor (κ 0.18).

**Fig. 2:**
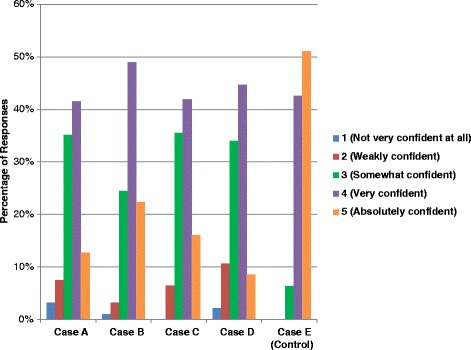
Level of confidence in sepsis diagnoses for each case

In the subset of respondents who were strongly confident (*n* = 45) in their ability to describe and use sepsis definitions, agreement was no better (κ 0.28 for the five-level classification, κ 0.21 for the dichotomized severe sepsis/septic shock classification, and κ 0.21 for the dichotomized sepsis/severe sepsis/septic shock classification). Most respondents felt “somewhat confident” or “very confident” in their assignment of sepsis diagnoses in each case; for the control case (case E), most respondents were either “very confident” (42.6 %) or “absolutely confident” (51.1 %) in their diagnosis (Fig. 2). Collectively, respondents felt somewhat, very, or absolutely confident about 93.2 % of their diagnoses.

**Fig. 3:**
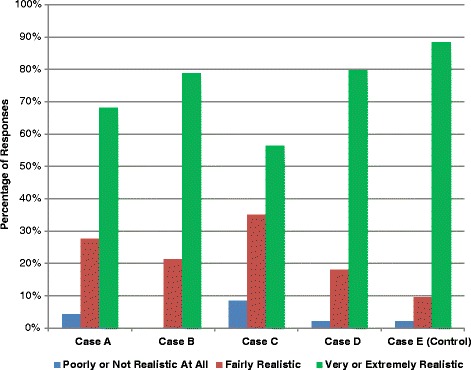
Qualitative assessment of case vignettes’ realism and representativeness of actual patients

Importantly, most respondents felt the cases to be realistic and representative of actual patients (Fig. 3). Of 470 ratings, 349 (74.3 %) were judged as “very realistic” or “extremely realistic.” Only 16 (3.4 %) were judged to be “poorly realistic” or “not realistic at all.”

### Reasons for not diagnosing severe sepsis/septic shock: areas of subjectivity

Respondents provided free text explanations for their decisions in 377 (80.2 %) of 470 of their diagnoses. For the cases labeled as not having severe sepsis or septic shock with an explanation (*n* = 172), virtually all explanations could be summarized into the following categories: no infection or organ dysfunction present (9.9 %), infection present but organ dysfunction not present or not severe enough to qualify as severe sepsis or septic shock (48.8 %), organ dysfunction present but no infection (18.6 %), and infection and organ dysfunction present but organ dysfunction not attributable to infection (22.1 %). The distribution of explanations differed for each case (Table 2). For case A, most of the disagreement centered on whether infection was present. For cases B–D, most of the disagreement centered on whether the patient had sufficient organ dysfunction or whether organ dysfunction was attributable to infection. Only one explanation was judged to represent a misunderstanding of the severe sepsis definition: the respondent incorrectly noted “SIRS and infection and signs of organ dysfunction = sepsis” rather than severe sepsis.

**Table 2:** Summary of explanations for why cases were not diagnosed as severe sepsis/septic shock

	No infection or organ dysfunction	Infection but organ dysfunction not present or not severe enough to qualify	Organ dysfunction but no infection	Infection and organ dysfunction, but not attributable to infection	Misclassified^a^	No explanation given	Representative quotes
Case A (pneumonia, heart failure, respiratory failure, and shock)	0	0	23	16	0	9	“Sounds like cardiogenic shock. Although heart rate and white blood cell count would meet SIRS criteria, this does not appear to be inflammatory in etiology. The mixed organisms are unconvincing for a true infection.”
Case B (pyelonephritis, acute kidney injury)	0	36	0	2	0	10	“SIRS (3/4 – temperature, heart rate, and white blood cell count) plus documented infection so sepsis. Although she had some degree of renal dysfunction, it resolved with fluids and antibiotics, so I would not classify her as severe sepsis.”
Case C (colitis, hypotension)	8	22	4	11	1	14	“She’s tachycardic, has low grade fever, hypotension, mild acute kidney injury, and CT evidence of colitis, so this seems related to infection, but she gets better with fluids and antibiotics quickly, with a high normal lactate that normalized rapidly, so no shock.”
Case D (COPD exacerbation, respiratory failure)	9	26	5	9	0	16	“COPD exacerbation with suspected source of infection, but no hypotension or lactate elevation.”

*COPD* chronic obstructive pulmonary disease, *CT* computed tomography, *SIRS* systemic inflammatory response syndrome

^a^ “Misclassified” refers to case that was not labeled as severe sepsis/septic shock but explanation was indicative of severe sepsis

## Discussion

In this survey of 94 physicians, who primarily were attending intensivists at academic institutions, we found poor agreement in diagnosing sepsis, severe sepsis, or septic shock when respondents were presented with short clinical case vignettes. For purposes of quality monitoring, it is more meaningful to determine whether patients had severe sepsis/septic shock. However, when we examined responses dichotomized in this way, agreement was no better. In addition, when the analysis was limited to physicians who were strongly confident in their ability to describe and apply the traditional international consensus definitions of sepsis, agreement remained poor. Importantly, these fictional vignettes were generally felt to be very realistic and representative of common clinical scenarios.

To our knowledge, this is the first study to examine variability in diagnosing sepsis by presenting identical cases to a group of intensivists. In an international qualitative survey of over 1000 physicians (including 529 intensivists) performed in 2000 by telephone interview, researchers found that less than 20 % of respondents gave a consistent definition of sepsis, with many physicians having the misperception that fever or hypotension must be present to diagnose sepsis [14]. However, since that survey was done, there have been substantial advances in sepsis awareness due to international initiatives such as the Surviving Sepsis Campaign, the dissemination of evidence-based management guidelines for sepsis, the publication of many high-profile clinical studies, and the recent introduction of national mandates for sepsis care and public reporting [15–17]. Our findings suggest that, even with the increased awareness and focus on sepsis in recent years, there is still a significant amount of variability in diagnosing sepsis among critical care physicians—the specialists who are generally felt to have the most expertise in caring for patients with sepsis.

Subjectivity in diagnosing sepsis is to be expected early in a patient’s clinical course, when symptoms are undifferentiated and diagnostic test results are still pending. However, in our study, we used a case vignette format in which the patient’s entire clinical course was presented. We nonetheless found substantial variability in how sepsis diagnoses were assigned. Although we did not explicitly test respondents’ knowledge of the sepsis definitions, in our analysis of free text explanations we found that variability was generally due to differences in interpreting whether infection or organ dysfunction was present or if organ dysfunction was attributable to infection, rather than to a lack of knowledge about the meaning of the sepsis definitions. Prior studies have suggested that adult and pediatric clinicians often disagree about sepsis diagnoses when compared with rigorous application of international consensus definitions; however, the researchers in these studies presumed that the consensus definitions themselves can be consistently applied [18, 19]. Interestingly, even when dichotomizing responses into any sepsis category (sepsis, severe sepsis, or septic shock) or not, there was still substantial disagreement in our study, indicating that simply deciding whether a patient has an infection can be highly variable, even in retrospect. This is important when considering the new consensus clinical definitions for sepsis recently released by the Society of Critical Care Medicine (SCCM) and the European Society of Intensive Care Medicine (ESICM) [20]. Although the terminology and criteria for organ dysfunction are being updated, this new definition still relies on clinicians’ judgement of whether infection is present, as well as whether organ dysfunction is attributable to infection.

Our findings have important implications for epidemiologic studies, public health surveillance, and quality reporting. Currently, the method proposed by the Centers for Medicare and Medicaid Services (CMS) for identifying cases of severe sepsis for reporting of sepsis bundle adherence is based on International Classification of Diseases, Tenth Revision, codes for sepsis, followed by chart review [4]. However, relying on diagnoses and codes is problematic when it comes to identifying sepsis cases and outcomes, as our study demonstrates that there is wide variability between clinicians in how they diagnose sepsis. This complicates current initiatives to benchmark hospitals on their care of patients with sepsis, since there is no common standard, it seems, for what constitutes a “septic” patient. Using claims data for longitudinal surveillance of sepsis trends is also complicated by the fact that the ways in which clinicians and hospitals diagnose and code for sepsis are changing over time, likely in response to rising awareness of sepsis and changing reimbursement incentives [3, 21–23]. Prior studies have shown that incorporating quality metrics and potential financial penalties for conditions where there is substantial room for subjectivity in diagnosis, such as ventilator-associated pneumonia, can lead to misleading decreases in disease incidence that better reflect stricter application of subjective diagnostic criteria rather than true reductions in the number of cases [24]. One alternative approach to surveillance that has recently been proposed is to use an objective surveillance definition that relies on electronically ascertainable clinical markers of presumed infection (such as blood cultures and antibiotics) and concurrent organ dysfunction (such as vasopressors, mechanical ventilation, and standardized changes in baseline laboratory values) rather than subjective and variable diagnoses and claim codes [23]. This approach will increase objectivity and reproducibility, although it does not solve the problem of knowing with certainty whether a patient is infected and whether concurrent organ dysfunction is attributable to infection.

While our study suggests substantial interobserver variability in diagnosing sepsis, it is important to note that several research studies have shown reasonable to good agreement (with κ statistics in the 0.6–0.8 range) among physicians using chart reviews as a gold standard for identifying severe sepsis [23, 25, 26]. However, the raters in these studies were formally trained using shared sets of cases, used standardized abstraction tools, and deliberately attempted to reconcile discrepant results. Intensive training, standardized abstraction tools, and formal reconciliation conferences are not part of routine clinical or coding practices, and hence the lower levels of agreement we observed in the present study may be more representative of real-world practice than the high levels of agreement reported in research studies. In addition, agreement about whether sepsis is present is likely to be higher in “sentinel” cases. For example, one prior study showed that greater severity of illness, ICU admission, bacteremia, elevated lactate, and shock were associated with greater consistency in the diagnosis of severe sepsis [27]. However, clear-cut cases with severe illness and unambiguous infection—similar to the control case we used in our study—represent only a small subset of sepsis cases.

Our study has important limitations. First, the response rate of our survey was relatively low, and we were unable to compare the characteristics of physicians who were contacted but did not respond. However, if anything, we would expect that physicians who completed the survey might have a greater degree of interest in (and knowledge about) sepsis than nonrespondents. It is thus even possible that this could have biased our results to overestimate agreement. Second, our survey was heavily weighted toward academic physicians in the northeastern United States, limiting the generalizability of our findings. Third, it is possible that overall agreement would be better in a large, random sample of actual patients with suspected infection. However, respondents in the present study generally felt that the study vignettes were both realistic and representative of actual patients, underscoring the fact that ambiguous cases of sepsis are likely fairly common. Fourth, our survey was conducted before the release of the new SCCM/ESICM consensus definition of sepsis, which may have performance characteristics in terms of interobserver variability that are different from those of the prior sepsis definition set. However, the new definition uses the same framework of seeking patients with acute organ dysfunction attributable to infection, and hence subjectivity in assigning sepsis diagnoses will likely persist. Furthermore, it may take time for these new definitions to gain full acceptance in the medical community, while the traditional definitions will still be used for the foreseeable future as part of the CMS quality metrics.

## Conclusions

Assignment of sepsis diagnoses is extremely variable, even among intensivists who report being very familiar with sepsis definitions and confident in their ability to apply them. This has important implications for the interpretation of sepsis quality improvement initiatives and the CMS sepsis bundle adherence reporting requirement, as well as for epidemiologic studies and clinical trials. More objective criteria and standardized methodology are needed to enhance consistency and comparability in sepsis research, surveillance, and quality reporting.

## Key messages

- Interobserver agreement among intensivists in diagnosing sepsis is poor.
- When diagnosing sepsis, there is a substantial amount of subjectivity in deciding whether infection is present, whether acute organ dysfunction is present, and whether acute organ dysfunction is attributable to infection.
- Subjectivity in diagnosing sepsis must be taken into account when interpreting the results of sepsis quality improvement initiatives and public reporting for sepsis bundle adherence, as well as for epidemiologic studies and clinical trials.
- Objective criteria and standardized methodology are needed to enhance consistency and comparability in sepsis research, surveillance, and quality reporting.

## Additional file

Additional file 1: Appendix 1. Online survey questions and case vignettes. (DOCX 31 kb)

## Abbreviations

CMS: Centers for Medicare & Medicaid Services
COPD: chronic obstructive pulmonary disease
CT: computed tomography
ESICM: European Society of Intensive Care Medicine
ICU: intensive care unit
IQR: interquartile range
SCCM: Society of Critical Care Medicine
SIRS: systemic inflammatory response syndrome
USCIITG: U.S. Critical Illness and Injury Trials Group
