# Supplementary material for: Diagnosing sepsis is subjective and highly variable: a survey of intensivists using case vignettes
Source: Crit Care. 2016 Apr 6;20:89. doi: 10.1186/s13054-016-1266-9 (PMC4822273; doi:10.1186/s13054-016-1266-9)
Supplement: Additional file 1: — Appendix 1. Online survey questions and case vignettes. (DOCX 31 kb) [file 13054_2016_1266_MOESM1_ESM.docx]

1. **INTRODUCTION**

The purpose of this study is to examine how physicians, particularly intensivists, apply sepsis diagnoses to patients.

After several simple background questions, there will be **FIVE** case vignettes, with questions asking your opinion about sepsis classifications.  For each vignette, for laboratory studies that are not explicitly provided, please assume that all others are within normal limits.

Responses will not be linked to individual respondents or their institutions, and results will only be presented in de-identified aggregate form.

Thank you very much in advance for your participation!

1. **BACKGROUND QUESTIONS**
2. What is your specialty? Choose all that apply.

- Anesthesia
- Cardiology
- Critical Care
- Emergency Medicine
- Infectious Diseases
- General Internal Medicine
- Nephrology
- Pediatrics
- Pulmonology
- Surgery
- Neurology
- Other (Please Specify):

1. What percentage of your professional time do you spend taking care of patients (i.e., what percentage of your time is “clinical time”?
2. What percentage of your clinical time is spent taking care of ICU patients?
3. How many adult ICU patients, on average, do you administer care to in one month?
4. How many years have you been in practice after completing your postgraduate training?
5. Which of the following best describes the hospital where you spend most of your clinical time?

- Community Hospital, rural or suburban
- Community Hospital, urban
- Academic Hospital, rural or suburban
- Academic Hospital, urban

1. Please describe the geographic region where you practice.

- Northeast (U.S.)
- Midwest (U.S.)
- South (U.S.)
- West (U.S.)
- Non-U.S. Country

1. Please state your level of agreement or disagreement with the following statement: “I am confident in my ability to describe and use the international consensus clinical definitions of sepsis, severe sepsis, and septic shock.”

- 1 - Strongly disagree
- 2 - Somewhat disagree
- 3 - Neither agree nor disagree
- 4 - Somewhat agree
- 5 - Strongly agree

1. **CASE VIGNETTES**

**Case A**

67 year old male with severe ischemic cardiomyopathy (ejection fraction 20%) presents with several days of shortness of breath, orthopnea, lower extremity edema, malaise, productive cough, and fevers. 

**ER vitals/ notable exam findings:** Temperature 100.2° F, Heart rate 145 (atrial fibrillation), Blood pressure 90/65 mmHg, Respiratory rate 24, Oxygen Saturation 88% on room air. Jugular veins distended, (+) peripheral edema, scattered rales throughout lungs. 

**Labs/Studies:** WBC 12.5 K/µL (no bands), Lactate 2.1 mmol/L, Creatinine 1.3 mg/dL (baseline 1.0). Chest X-ray shows mild pulmonary edema and a left lower lobe infiltrate.

**ER course:** Given 1 liter of normal saline, IV levofloxacin, and 5 mg diltiazem IV for rapid a-fib. Blood pressure drops to 80/60, noted to have cool extremities, repeat lactate 4.1. Develops worsening hypoxemia and altered mental status and is intubated. Central line placed, started on norepinephrine and dobutamine. Initial central venous pressure 16 cm H20 and central venous oxygen saturation 48%. Admitted to ICU.

**ICU course:** Continued on norepinephrine and dobutamine as well as amiodarone for rate control. Gently diuresed with furosemide drip. Antibiotics broadened to include vancomycin and cefepime. Sputum cultures grow mixed respiratory flora; blood cultures are negative. Lactate and WBC normalize; urine output and creatinine gradually improve. Extubated on ICU day 3 and transitioned off vasoactive agents on ICU day 4. Completes 7 day course of antibiotics and discharged on hospital day 10.

1. How would you classify this patient’s clinical course?

- Systemic Inflammatory Response Syndrome (SIRS)
- Sepsis
- Severe Sepsis
- Septic Shock
- None of the above

1. Please briefly explain your rationale, in no more than one or two sentences:
2. Please rate the degree of certainty or confidence in your classification:

- 1 - Not confident at all
- 2 - Weakly confident
- 3 - Somewhat confident
- 4 - Very confident
- 5 - Absolutely confident

1. Please rate the degree to which you feel this case resembles a realistic scenario you have encountered or may actually encounter in the hospital.

- 1 - Not realistic at all, this does not resemble any patients I have ever cared for
- 2 - Poorly realistic, this resembles only a handful of patients I have cared for
- 3 - Fairly realistic, it resembles patients I have occasionally seen in the past
- 4 - Very realistic, it resembles patients I have seen quite often in the past
- 5 - Extremely realistic, I have cared for many patients with similar features in the past

**Case B**

29 year old female with a history of recurrent urinary tract infections presents to the ER with 4 days of progressive dysuria, left flank pain, nausea, vomiting, anorexia, and fevers, unresponsive to oral ciprofloxacin started as an outpatient 3 days ago. 

**ER vitals/ notable exam findings:** Temperature 101.7° F, Heart rate 135, Blood pressure 98/55 mmHg, Respiratory rate 16, Oxygen saturation 97% on room air. Left costovertebral tenderness.

**Labs/Studies:** WBC 13.2 K/µL (5% bands), Lactate 1.6 mmol/L, Blood urea nitrogen 50 mg/dL, Creatinine 1.3 mg/dL (baseline 0.8). Urinalysis floridly positive with WBC’s, leukocyte esterase, nitrites, and bacteria.

**ER Course:** Given 3 liters normal saline, started on IV cefepime, acetaminophen, and antiemetics. Blood pressure increases to 115/70, heart rate decreases to 95, and temperature decreases to 99.5. ICU initially consulted, but decision made to admit to medical ward.

**Hospital course:** Continued on IV fluids and cefepime. Nausea and anorexia improve. Urine culture grows E.coli, resistant to ciprofloxacin but sensitive to ceftriaxone and cefepime. Blood cultures remain negative. Creatinine improves to 1.1 on hospital day 1 and 0.8 on hospital day 2. Discharged on a 10 day course of cefpodoxime.

1. How would you classify this patient’s clinical course?

- Systemic Inflammatory Response Syndrome (SIRS)
- Sepsis
- Severe Sepsis
- Septic Shock
- None of the above

1. Please briefly explain your rationale, in no more than one or two sentences:
2. Please rate the degree of certainty or confidence in your classification:

- 1 - Not confident at all
- 2 - Weakly confident
- 3 - Somewhat confident
- 4 - Very confident
- 5 - Absolutely confident

1. Please rate the degree to which you feel this case resembles a realistic scenario you have encountered or may actually encounter in the hospital.

- 1 - Not realistic at all, this does not resemble any patients I have ever cared for
- 2 - Poorly realistic, this resembles only a handful of patients I have cared for
- 3 - Fairly realistic, it resembles patients I have occasionally seen in the past
- 4 - Very realistic, it resembles patients I have seen quite often in the past
- 5 - Extremely realistic, I have cared for many patients with similar features in the past

**Case C**

53 year old female with no medical history presents with 3 days of profuse watery diarrhea (>8-10 episodes/day), abdominal cramps, nausea, vomiting, and low grade fevers. She returned 4 days ago from a trip to Mexico where she ate all local foods and drank tap water. She has been taking bismuth subsalicylate (Pepto-Bismol) without improvement.

**ER vitals/ notable exam findings:** Temperature 100.5° F, Heart rate 130, Blood pressure 80/40 mmHg, Respiratory rate 12, Oxygen saturation 96% on room air. Moderate left lower quadrant tenderness to palpation without peritoneal signs. Decreased skin turgor.

**Labs:** WBC 12.1 K/µL (5% bands), Potassium 2.9 meq/L, Blood urea nitrogen 48 mg/dL, Creatinine 1.1 mg/dL (baseline 0.7), Lactate 2.0 mmol/L.

**ER course:** Given 2.5 liters of normal saline and potassium repletion. Blood cultures drawn, started on IV ciprofloxacin and metronidazole. Blood pressure increases to 110/60 and heart rate decreases to 92. Noncontrast abdominal CT scan shows mild wall thickening of the transverse and distal colon. Repeat lactate is 1.1. ICU initially consulted, but decision made to admit to the medical ward.

**Hospital course:** Creatinine 0.8 on the day following admission. Blood cultures, stool cultures, and Clostridium difficile tests are negative. Diarrhea gradually improves. Discharged on hospital day 3 to complete a 7 day course of ciprofloxacin and metronidazole.

1. How would you classify this patient’s clinical course?

- Systemic Inflammatory Response Syndrome (SIRS)
- Sepsis
- Severe Sepsis
- Septic Shock
- None of the above

1. Please briefly explain your rationale, in no more than one or two sentences:
2. Please rate the degree of certainty or confidence in your classification:

- 1 - Not confident at all
- 2 - Weakly confident
- 3 - Somewhat confident
- 4 - Very confident
- 5 - Absolutely confident

1. Please rate the degree to which you feel this case resembles a realistic scenario you have encountered or may actually encounter in the hospital.

- 1 - Not realistic at all, this does not resemble any patients I have ever cared for
- 2 - Poorly realistic, this resembles only a handful of patients I have cared for
- 3 - Fairly realistic, it resembles patients I have occasionally seen in the past
- 4 - Very realistic, it resembles patients I have seen quite often in the past
- 5 - Extremely realistic, I have cared for many patients with similar features in the past

**Case D**

70 year old male with severe COPD presents with 3 days of progressive shortness of breath, wheezing, productive cough with purulent sputum, and subjective fevers. He was recently hospitalized for a COPD exacerbation and just finished a prednisone taper 4 days ago.

**ER vitals/ notable exam findings:** Temperature 101.4° F, Heart rate 130, Blood pressure 103/60, Respiratory rate 36, Oxygen saturation 85% on room air. Decreased breath sounds and wheezing.

**Labs:** WBC 11.0 K/µL (no bands), Creatinine 0.9 mg/dL, Lactate 0.9 mmol/L. Chest x-ray shows hyperinflated lungs, no consolidation. Arterial blood gas: pH 7.05, pCO2 90 mmHg, pO2 50 mmHg.

**ER course:** Given IV methylprednisolone, albuterol and ipratropium nebulizers, IV vancomycin and cefepime, and started on noninvasive positive pressure ventilation. Respiratory distress worsens and he is urgently intubated. CT angiogram shows no pulmonary embolism and no consolidation.

**Hospital Course:** Remains intubated for 3 days; continued on steroids, bronchodilators, and vancomycin and cefepime. Fevers resolve, blood pressure remains stable. Blood cultures are negative, sputum grows H.influenza. Chest x-ray remains clear. Extubated on ICU day 4. Completes a 7 day course of levofloxacin and is discharged to rehab on hospital day 10.

1. How would you classify this patient’s clinical course?

- Systemic Inflammatory Response Syndrome (SIRS)
- Sepsis
- Severe Sepsis
- Septic Shock
- None of the above

1. Please briefly explain your rationale, in no more than one or two sentences:
2. Please rate the degree of certainty or confidence in your classification:

- 1 - Not confident at all
- 2 - Weakly confident
- 3 - Somewhat confident
- 4 - Very confident
- 5 - Absolutely confident

1. Please rate the degree to which you feel this case resembles a realistic scenario you have encountered or may actually encounter in the hospital.

- 1 - Not realistic at all, this does not resemble any patients I have ever cared for
- 2 - Poorly realistic, this resembles only a handful of patients I have cared for
- 3 - Fairly realistic, it resembles patients I have occasionally seen in the past
- 4 - Very realistic, it resembles patients I have seen quite often in the past
- 5 - Extremely realistic, I have cared for many patients with similar features in the past

**Case E**

61 year old female is hospitalized with recurrent acute myelogenous leukemia and receives induction chemotherapy. One week after completing chemotherapy, she is neutropenic, and while on the wards she develops abrupt onset of fevers, rigors, and shortness of breath.

**Floor vitals/notable exam findings:** T 103.1° F, Heart rate 145, Blood pressure 75/30 mmHg, Respiratory rate 32, Oxygen saturation 90% on room air. Tachypneic and confused.

**Labs:** WBC 0 K/µL, Platelets 10 K/µL, Creatinine 1.1 mg/dL (baseline 0.7), Lactate 4.1 mmol/L. Chest x-ray shows bilateral patchy opacities.

**Floor/ICU course:** Blood cultures drawn, started on IV vancomycin and cefepime, given 4 liters of normal saline and IV acetaminophen. Blood pressure remains low, repeat lactate is 5.0. Transferred to medical ICU where she is intubated due to worsening hypoxia and somnolence, and started on norepinephrine for persistent hypotension. A central venous catheter is placed and her tunneled central venous catheter is removed. Blood cultures grow Gram-negative rods in 4 out of 4 bottles, eventually speciated as Pseudomonas aeruginosa. By ICU day 3 she is on four vasopressors and maximal ventilator settings, and develops anuric renal failure. She expires on ICU day 4.

1. How would you classify this patient’s clinical course?

- Systemic Inflammatory Response Syndrome (SIRS)
- Sepsis
- Severe Sepsis
- Septic Shock
- None of the above

1. Please briefly explain your rationale, in no more than one or two sentences:
2. Please rate the degree of certainty or confidence in your classification:

- 1 - Not confident at all
- 2 - Weakly confident
- 3 - Somewhat confident
- 4 - Very confident
- 5 - Absolutely confident

1. Please rate the degree to which you feel this case resembles a realistic scenario you have encountered or may actually encounter in the hospital.

- 1 - Not realistic at all, this does not resemble any patients I have ever cared for
- 2 - Poorly realistic, this resembles only a handful of patients I have cared for
- 3 - Fairly realistic, it resembles patients I have occasionally seen in the past
- 4 - Very realistic, it resembles patients I have seen quite often in the past
- 5 - Extremely realistic, I have cared for many patients with similar features in the past
